# Inhibition of mTORC1 through ATF4-induced REDD1 and Sestrin2 expression by Metformin

**DOI:** 10.1186/s12885-021-08346-x

**Published:** 2021-07-12

**Authors:** Se-Kyeong Jang, Sung-Eun Hong, Da-Hee Lee, Ji-Young Kim, Ji Yea Kim, Sang-Kyu Ye, Jungil Hong, In-Chul Park, Hyeon-Ok Jin

**Affiliations:** 1 Division of Fusion Radiology Research, Korea Institute of Radiological & Medical Sciences, 75 Nowon-ro, Nowon-gu, Seoul, 01812 Republic of Korea; 2 Department of Food and Microbial Technology, Seoul Women’s University, 621 Hwarangro, Nowon-gu, Seoul, 01797 Republic of Korea; 3 KIRAMS Radiation Biobank, Korea Institute of Radiological and Medical Sciences, 75 Nowon-ro, Nowon-gu, Seoul, 01812 Republic of Korea; 4 Department of Pharmacology, Seoul National University College of Medicine, 103 Daehak-ro, Jongno-gu, Seoul, 03080 Republic of Korea

**Keywords:** AMPK, Metformin, mTORC1, REDD1, Sestrin2

## Abstract

**Background:**

Although the major anticancer effect of metformin involves AMPK-dependent or AMPK-independent mTORC1 inhibition, the mechanisms of action are still not fully understood.

**Methods:**

To investigate the molecular mechanisms underlying the effect of metformin on the mTORC1 inhibition, MTT assay, RT-PCR, and western blot analysis were performed.

**Results:**

Metformin induced the expression of ATF4, REDD1, and Sestrin2 concomitant with its inhibition of mTORC1 activity. Treatment with REDD1 or Sestrin2 siRNA reversed the mTORC1 inhibition induced by metformin, indicating that REDD1 and Sestrin2 are important for the inhibition of mTORC1 triggered by metformin treatment. Moreover, REDD1- and Sestrin2-mediated mTORC1 inhibition in response to metformin was independent of AMPK activation. Additionally, lapatinib enhances cell sensitivity to metformin, and knockdown of REDD1 and Sestrin2 decreased cell sensitivity to metformin and lapatinib.

**Conclusions:**

ATF4-induced REDD1 and Sestrin2 expression in response to metformin plays an important role in mTORC1 inhibition independent of AMPK activation, and this signalling pathway could have therapeutic value.

## Background

Metformin (1,1-dimethylbiguanide hydrochloride) belongs to the biguanide class of drugs and is a widely used drug administered orally to treat type 2 diabetes mellitus [1]. Epidemiological studies have demonstrated that metformin use is associated with decreased cancer incidence and mortality in patients with diabetes [2, 3]. Furthermore, accumulating evidence suggests that metformin exerts antitumour effects in many cancers [4–6]. However, the underlying molecular mechanism by which metformin reduces tumour incidence and inhibits cancer cell growth in vitro and in vivo has not been clearly elucidated. The well-accepted mechanism of metformin action is inhibition of mitochondrial respiratory complex I and activation of AMP-activated protein kinase (AMPK) in response to energy depletion [7]. AMPK is a heterodimeric protein complex that plays an essential role in sensing energy and suppressing cell growth under low-energy conditions [8]. Activated AMPK phosphorylates multiple downstream targets to maintain cellular energy homeostasis [8, 9].

One of the central downstream targets inhibited by AMPK is mechanistic target of rapamycin complex 1 (mTORC1), a serine/threonine kinase that has a critical role in controlling cell growth and cellular metabolism by integrating various environmental signals, such as growth factors, amino acids, and glucose [10, 11]. mTORC1 directly phosphorylates downstream substrates, including ribosomal S6 kinase 1 (S6K1) and eukaryotic initiation factor 4E (eIF4E)-binding protein 1 (4E-BP1), to regulate protein synthesis to promote cell proliferation [12]. mTORC1 is tightly regulated by multiple upstream pathways. The response of mTORC1 signalling to growth factors is mediated by the small GTPase Ras homology enriched in brain (Rheb), which is negatively regulated by tuberous sclerosis complex (TSC1/2) proteins [13–15]. When the PI3K/Akt pathway is activated by growth factors, Akt phosphorylates TSC2 and disrupts the TSC1/2 complex [16, 17]. Energy levels signal to mTORC1 through AMPK by two mechanisms [18]. Firstly, AMPK directly phosphorylates the TSC2 on S1387 to activate TSC2 and promote inhibition of mTORC1 inhibition through the Rheb axis [19, 20]. The second, AMPK phosphorylates raptor on serines 722 and 792 to directly inhibit mTORC1 activity [21]. Some studies have reported that metformin inhibits the mTORC1 signalling pathway independent of AMPK activation [22–24]. However, the molecular mechanisms involved in AMPK-independent mTORC1 inhibition by metformin have not been fully elucidated.

In the present study, we investigated the molecular mechanism(s) by which metformin induces mTORC1 inhibition in non-small cell lung cancer (NSCLC) cells. We found that inhibition of mTORC1 in response to metformin requires ATF4 and that ATF4-induced upregulation of REDD1 and Sestrin2 is implicated in this effect. REDD1 and Sestrin2 are necessary for mTORC1 inhibition by metformin treatment, and the response occurs through an ATF4-dependent mechanism in NSCLC cells. In conclusion, ATF4-induced REDD1 and Sestrin2 expression triggered by metformin plays an important role in mTORC1 inhibition independent of AMPK activation.

## Methods

### Cell culture and reagents

H1299 NSCLC cells were obtained from ATCC (Manassas, VA, USA) and cultured in RPMI 1640 medium (#LM011–01; Welgene, Gyeongsangbuk-do, Republic of Korea) supplemented with 10% foetal bovine serum (Gibco; Thermo Fisher Scientific, Waltham, MA, USA). Metformin, phenformin and thiazolyl blue tetrazolium bromide (MTT) were purchased from Sigma-Aldrich (Merck KGaA, Darmstadt, Germany).

### Cell viability assay

Cell viability was assessed by measuring the mitochondrial conversion of MTT. The proportion of converted MTT was calculated by measuring the absorbance at 570 nm. The results are expressed as the percentage reduction in MTT under the assumption that the absorbance of the control cells was 100%. The MTT experiments were repeated three times.

### RNA extraction and reverse transcription polymerase chain reaction (RT-PCR)

RNA was isolated from H1299 cells using TRIzol Reagent according to the manufacturer’s instructions (Invitrogen; Thermo Fisher Scientific). cDNA primed with oligo dT was prepared from 2 μg total RNA using M-MLV Reverse Transcriptase (Invitrogen; Thermo Fisher Scientific).

The following specific primers were used for PCR: *ATF4:* 5′-AGTCGGGTTTGGGGGCTGAAG − 3′ and 5′-TGGGGAAAGGGGAAGAGGTTGTAA-3′, 437 bp product; *β-actin*: 5′-GGATTCCTATGTGGGCGACAG-3′ and 5′-CGCTCGGTGAGGATCTTCATG-3′, 438 bp product. The PCR products were visualized on a 2% agarose gel containing ethidium bromide.

### Real-time PCR

Real-time PCR was conducted using TaqMan Gene Expression Assay Probes (Applied Biosystems, Foster City, CA, USA) for mRNA quantification of *Redd1* (assay ID: Hs01111681_g1), *Sestrin1* (assay ID: Hs00902782_m1), *Sestrin2* (assay ID: Hs00230241_m1), and *Sestrin3* (assay ID: Hs00914870_m1). *β-actin* (assay ID: Hs01060665_g1) was used as an internal control. Quantitative real-time PCR was performed using an ABI 7500 Real-Time PCR System (Applied Biosystems). The fold change in gene expression was determined using the comparative CT (2^–ΔΔCT^) method.

### Transient transfection

ATF4 (#sc-35,112), REDD1 (#sc-45,806), Sestrin2 (sc-106,544) and control (#sc-37,007) siRNAs were purchased from Santa Cruz Biotechnology (Dallas, TX, USA). siRNA transfections in H1299 cells were performed using Lipofectamine RNAiMAX according to the manufacturer’s instructions (Invitrogen; Thermo Fisher Scientific).

### Western blot analysis

Proteins from cell lysates were separated using 6–11% sodium dodecyl sulphate-polyacrylamide gels and transferred to nitrocellulose membranes followed by immunoblotting with the specified primary and horseradish peroxidase-conjugated secondary antibodies. The following antibodies were used: S6K (#9202), p-S6K (Thr389) (#9205), 4E-BP1 (#9644), ACC (#3662), p-ACC (Ser79) (#3661), AMPKα (#2532), and p-AMPKα (Thr172) (#2535) were obtained from Cell Signaling Technology. The ATF4 (#sc-200) antibody was obtained from Santa Cruz Biotechnology. The REDD1 (#10638–1-AP), Sestrin1 (#21668–1-AP), Sestrin2 (#10795–1-AP), and Sestrin3 (#11431–2-AP) antibodies were obtained from the Proteintech Group (Chicago, IL, USA), and the β-actin (#A5316) antibody was obtained from Sigma-Aldrich (Merck KGaA).

### Statistical analysis

Data are expressed as the mean ± standard deviation (SD) of three independent experiments. Statistical analysis was performed using one-way analysis of variance followed by Tukey’s post hoc test with the GraphPad Prism software (Version 5.0; GraphPad Software Inc., San Diego, CA, USA). *P* < 0.05, *P* < 0.01 and *P* < 0.001 were considered to indicate statistically significant results.

## Results

### Metformin induces mTORC1 inhibition through AMPK activation

**Fig. 1:**
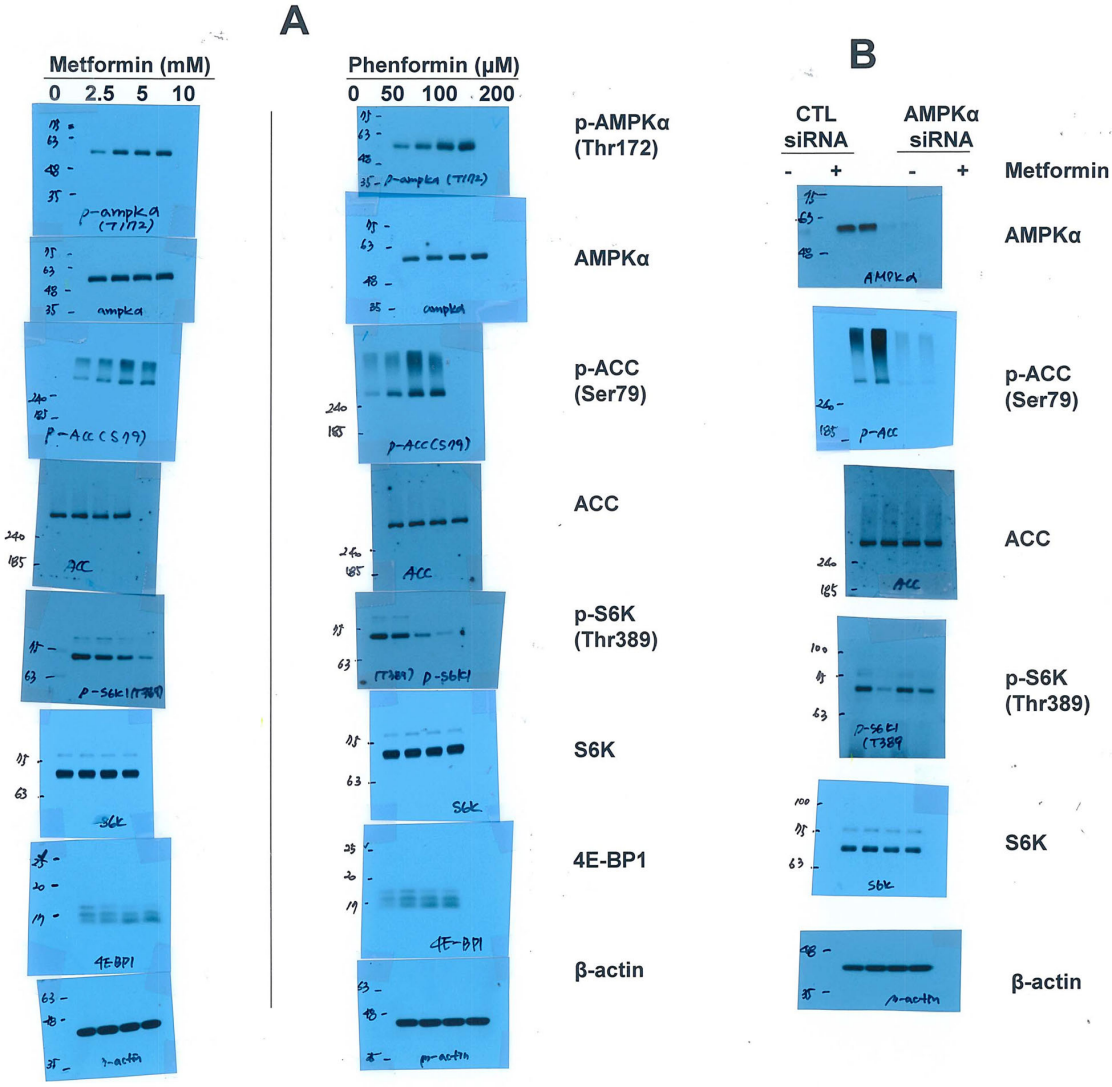
Metformin inhibits mTORC1 through AMPK. (a) H1299 cells were treated with the indicated concentrations of metformin or phenformin for 24 h. (b) H1299 cells were transfected with control or AMPKα siRNA for 12 h and were then treated with 10 mM metformin for 24 h. (a, b) Data are representative of two independent experiments. CTL: control

We first investigated the impact of metformin on mTORC1 activity in NSCLC cells. H1299 cells were treated with metformin at the above mentioned concentrations for 24 h. As shown in Fig. 1A, metformin inhibited mTORC1 activity, as shown by the decrease in S6K phosphorylation. Phosphorylation of 4E-BP1 was decreased by metformin, as evidenced by a shift to faster-migrating species [25]. Phenformin, a metformin analogue also inhibited mTORC1 activity, as assessed by reduced phosphorylation of S6K1 and 4E-BP1. It has been reported that metformin requires AMPK to inhibit mTORC1 [26]. As expected, metformin and phenformin both induced AMPK activation, as evaluated by the activating phosphorylation of Thr172 in AMPKα and Ser79 in the AMPK substrate acetyl-CoA carboxylase (ACC) (Fig. 1A). Next, we explored the effect of the absence of AMPK on metformin-induced mTORC1 inhibition. AMPKα siRNA abrogated AMPKα expression and prevented ACC phosphorylation induced by metformin treatment (Fig. 1B). The metformin-induced decrease in phfv.

osphorylated S6K was restored by knockdown of AMPKα (Fig. 1B). These data suggest that AMPK activation contributes to mTORC1 inhibition in response to metformin.

### Metformin induces mTORC1 inhibition through ATF4

**Fig. 2:**
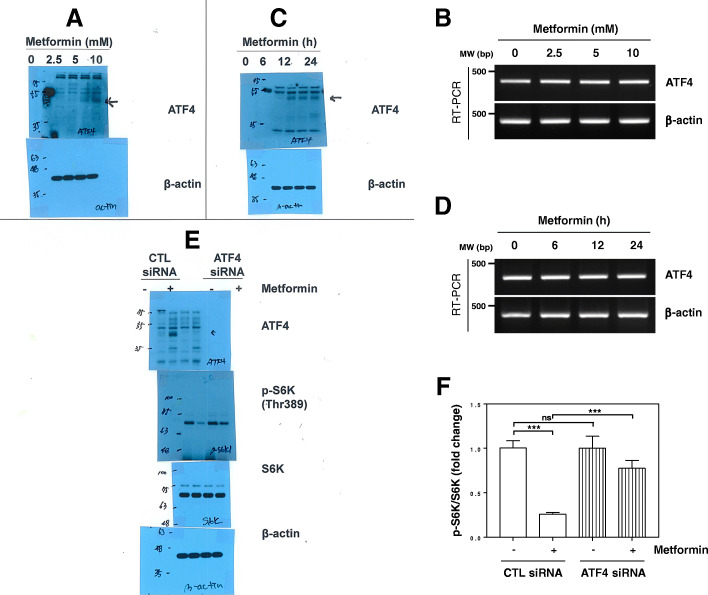
Metformin inhibits mTORC1 through ATF4. (a, b) H1299 cells were treated with the indicated concentrations of metformin for 24 h. (c, d) H1299 cells were treated with 10 mM metformin for the indicated time. The protein levels (a, c) and mRNA levels (b, d) were estimated by western blot and RT- PCR analysis, respectively. (a-d) Data are representative of two independent experiments. (e, f) H1299 cells were transfected with control or ATF4 siRNA for 12 h and were then treated with 10 mM metformin for 24 h. (e) Data are representative of three independent experiments. (f) The p-S6K expression was quantified using ImageJ software and fold change with respect to control after normalization to respective S6K bands was plotted as histogram. (*n* = 3; ***P < 0.001; NS, not significant). CTL: control

It has been reported that metformin can also inhibit mTORC1 through an AMPK-independent pathway [22–24]. Since activation of the PERK-eIF2α-ATF4 axis was triggered by metformin [27], we investigated whether ATF4 is involved in metformin-induced mTORC1 inhibition. Metformin induced ATF4 protein expression but did not affect the induction of ATF4 mRNA (Fig. 2A-D). The metformin-induced decrease in phosphorylated S6K was restored by knockdown of ATF4 (Fig. 2E and F), suggesting that ATF4 is necessary for metformin-mediated inhibition of mTORC1.

### REDD1 and Sestrin2 expression in the presence of metformin is regulated by ATF4

**Fig. 3:**
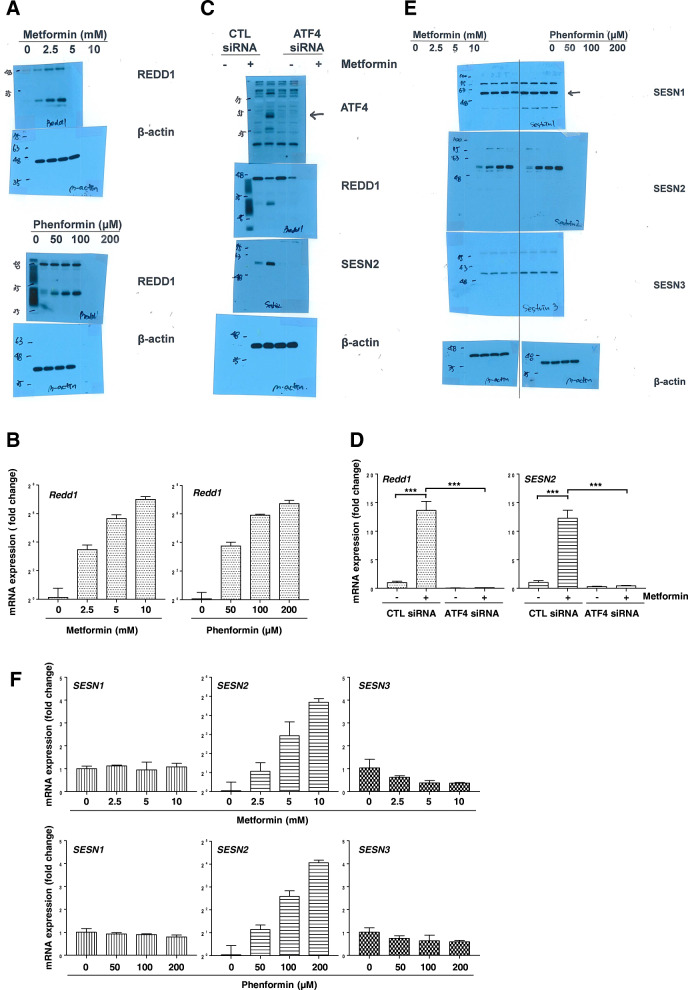
Metformin induces the expression of REDD1 and Sestrin2 in an ATF4-dependent manner. (a, b, e, f) H1299 cells were treated with the indicated concentrations of metformin or phenformin for 24 h. (c, d) H1299 cells were transfected with control or ATF4 siRNA for 12 h and were then treated with 10 mM metformin for 24 h. The protein levels (a, c, e) and mRNA levels (b, d, f) were estimated by western blot and real-time PCR analysis, respectively. (a, c, e) The western blot is representative of two independent experiments. (b, d, f) The real-time PCR results for each sample were analysed according to the 2^−ΔΔCt^ method using β-actin as the internal control. Gene transcription is presented as the fold change relative to the control sample (n = 3; ***P < 0.001). CTL: control, SESN1: Sestrin1, SESN2: Sestrin2, SESN3: Sestrin3

We previously reported that ATF4 facilitates the transcription of the REDD1 gene [28]. Thus, we investigated whether REDD1 expression is upregulated by metformin-induced ATF4 activation. Metformin and phenformin both induced REDD1 protein and mRNA expression in a dose-dependent manner (Fig. 3A and B). ATF4 siRNA almost completely blocked the upregulation of REDD1 in the presence of metformin (Fig. 3C and D). Sestrins are stress-inducible proteins that regulate metabolic homeostasis [29]. We investigated whether Sestrins are upregulated by metformin or phenformin. As shown in Fig. 3E and F, Sestrin2 protein and mRNA levels were upregulated under metformin or phenformin treatment. However, metformin and phenformin had no impact on the gene or protein expression of Sestrin1 or Sestrin3. We further investigated whether ATF4 is responsible for the upregulation of Sestrin2 expression in response to metformin. H1299 cells were transfected with ATF4 siRNA and treated with metformin. ATF4 siRNA blocked the upregulation of Sestrin2 in response to metformin (Fig. 3C and D). These data suggest that ATF4 activation is important for the induction of REDD1 and Sestrin2 expression by metformin treatment.

### AMPK and ATF4 do not affect each other’s expression in the presence of metformin

**Fig. 4:**
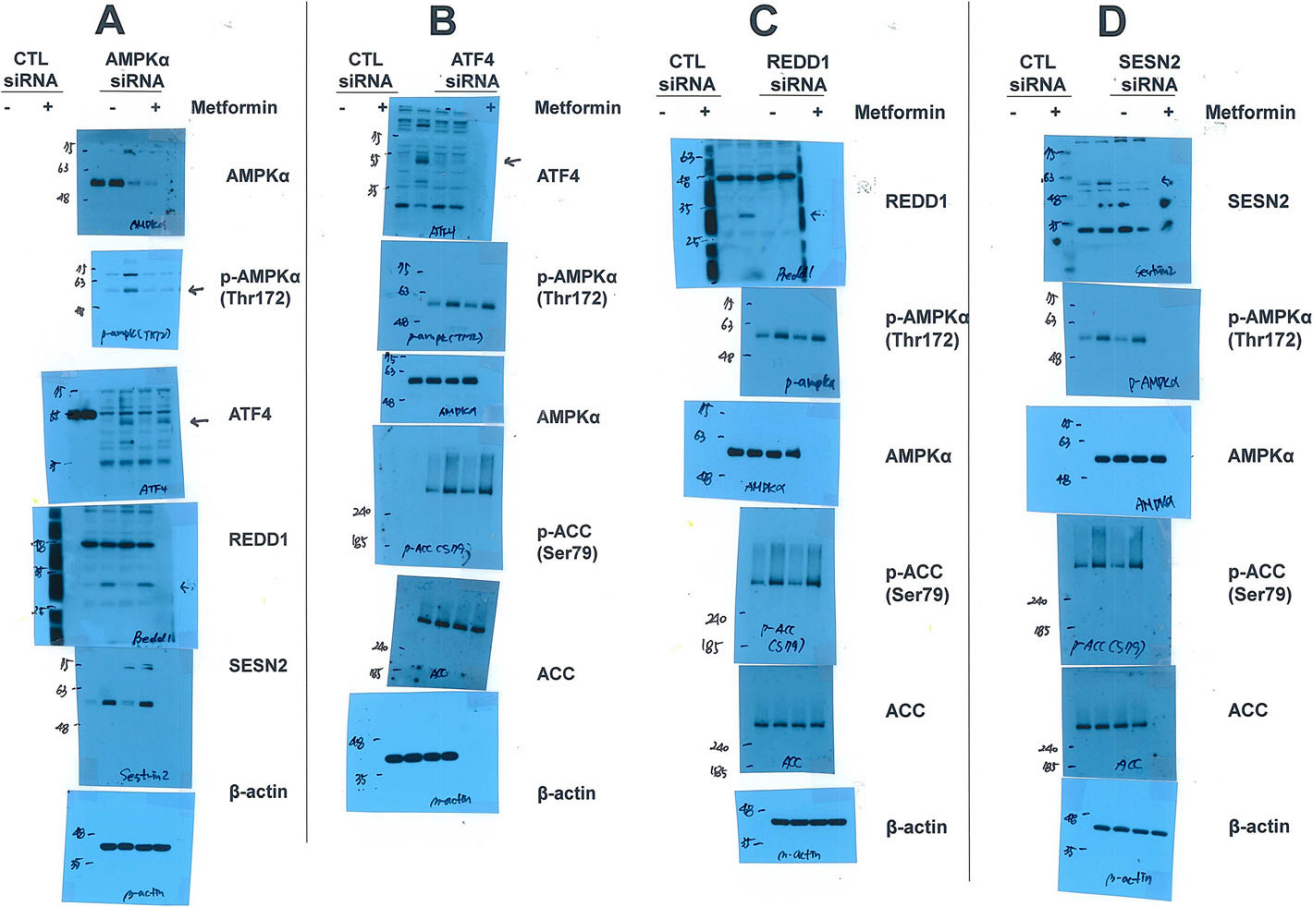
AMPK and ATF4 do not affect each other’s expression under metformin treatment. (a–d) H1299 cells were transfected with control, AMPKα, ATF4, REDD1, or Sestrin2 siRNA for 12 h and then treated with 10 mM metformin for 24 h. The indicated protein levels were estimated by western blot analysis. The blot is representative of two independent experiments. CTL: control, SESN2: Sestrin2

We investigated whether AMPK and ATF4 affect each other’s expression in the presence of metformin. We first investigated the protein expression of ATF4 and its downstream targets REDD1 and Sestrin2 by treatment with metformin in AMPK knockdown cells. The downregulation of AMPKα did not alter the induction of ATF4, REDD1 or Sestrin2 expression by metformin (Fig. 4A). Next, we investigated AMPK activation by metformin in cells with knockdown of ATF4 and its downstream targets REDD1 and Sestrin2. H1299 cells were transfected with ATF4, REDD1 and Sestrin2 siRNAs and then treated with metformin. Metformin-induced AMPKα and ACC phosphorylation was not changed by siRNAs against ATF4, REDD1 or Sestrin2 (Fig. 4B-D). These data suggest that AMPK and ATF4 do not affect each other’s expression in response to metformin.

### REDD1 and Sestrin2 expression induced by metformin is involved in mTORC1 inhibition

**Fig. 5:**
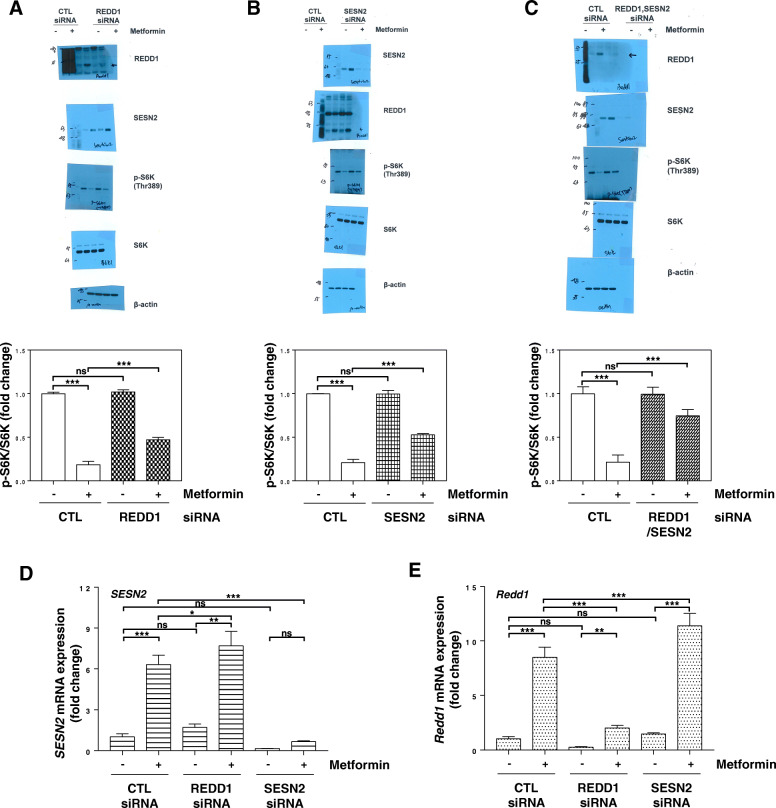
Metformin-induced REDD1 and Sestrin2 expression is involved in mTORC1 inhibition by metformin. (a-e) H1299 cells were transfected with control, REDD1, Sestrin2, or REDD1/Sestrein2 siRNA for 12 h and then treated with 10 mM metformin for 24 h. (a, b, c) The indicated protein levels were estimated by western blot analysis. (upper panels; a, b, c) The blot is representative of three independent experiments. (bottom panels; a, b, c) The p-S6K protein expression was quantified using ImageJ software and fold change with respect to control after normalization to respective S6K protein bands was plotted as histogram (*n* = 3; ***P < 0.001; ns, not significant). (d, e) The indicated mRNA levels were estimated by real-time PCR analysis. The real-time PCR results for each sample were analysed according to the 2^−ΔΔCt^ method using β-actin as the internal control. Gene transcription is presented as the fold change relative to the control sample (*n* = 3; *p < 0.05; **p < 0.01; ***p < 0.001; ns, not significant). CTL: control, SESN2: Sestrin2

We investigated whether REDD1 and/or Sestrin2 induction by metformin suppresses mTORC1 activity. As shown in Fig. 5A-C, the decreased phosphorylation of S6K by metformin was recovered in cells treated with REDD1 siRNA or Sestrin2 siRNA. Silencing both REDD1 and Sestrin2 further attenuated the effect of metformin on the inhibition of S6K phosphorylation. Interestingly, compared with that in the control siRNA-treated cells, the metformin-induced Sestrin2 expression was higher in the REDD1 siRNA-treated cells (Fig. 5A and D), and the metformin-induced REDD1 expression was higher in the Sestrin2 siRNA-treated cells (Fig. 5B and E). These data suggest that REDD1 and Sestrin2 are important for the inhibition of mTORC1 triggered by metformin treatment.

Lapatinib enhances cell sensitivity to metformin, and knockdown of REDD1 and Sestrin2 decreases cell sensitivity to metformin and lapatinib.

**Fig. 6:**
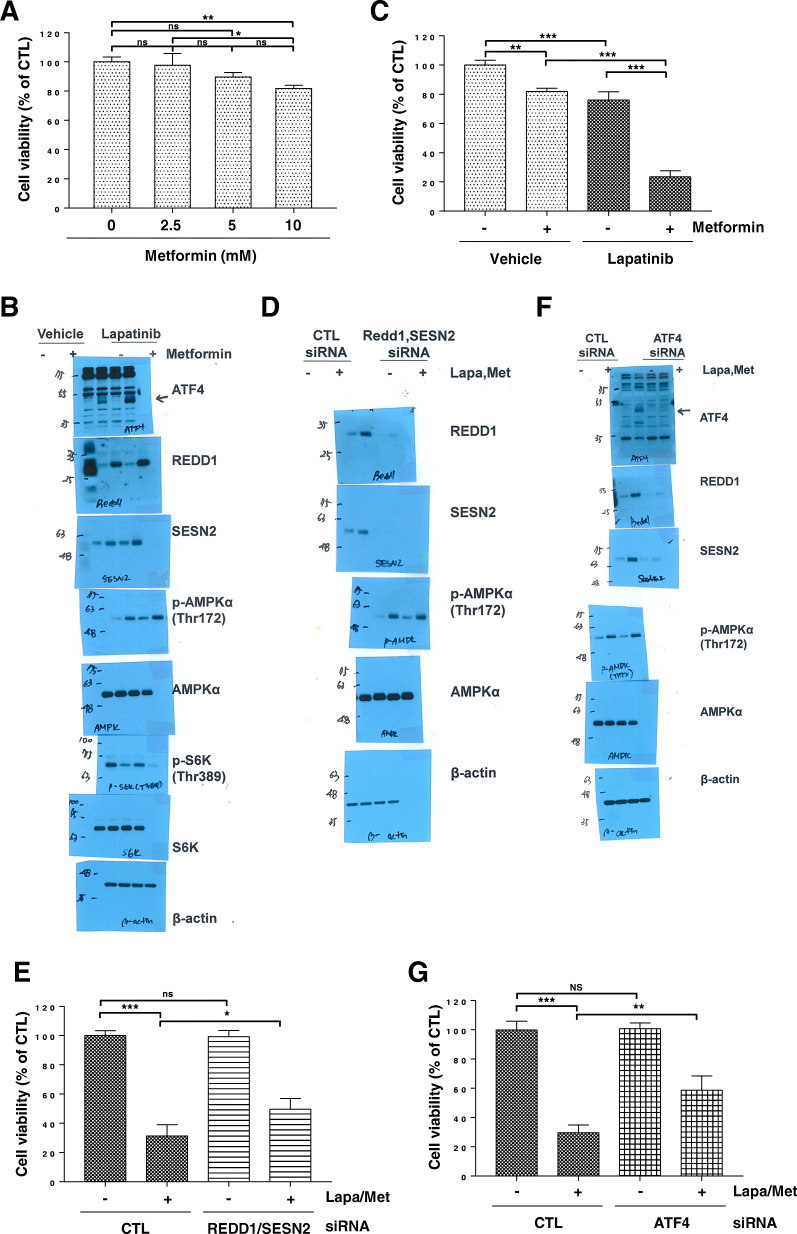
Lapatinib enhances cell sensitivity to metformin, and knockdown of REDD1 and Sestrin2 decreases cell sensitivity to metformin and lapatinib. (a) H1299 cells were treated with the indicated concentrations of metformin for 24 h. (b) H1299 cells were treated with 10 mM metformin and/or 10 μM lapatinib for 12 h. (c) H1299 cells were treated with 10 mM metformin and/or 10 μM lapatinib for 24 h. (d, f) H1299 cells were transfected with control, ATF4 or REDD1/Sestrin2 siRNA for 12 h followed by treatment with 10 mM metformin and 10 μM lapatinib for 12 h. (e, g) H1299 cells were transfected with control, ATF4, or REDD1/Sestrin2 siRNA for 12 h followed by treatment with 10 mM metformin and 10 μM lapatinib for 24 h. (a. c, e, g) Cell viability was measured by MTT assay. The data are presented as the mean percentage of control ± SD relative to the control (*n* = 3; *p < 0.05; **p < 0.01; ***p < 0.001; ns, not significantly different). (b, d, f) The indicated protein levels were estimated by western blot analysis. Data are representative of three independent experiments. CTL: control, Lapa: Lapatinib, Met: metformin, SESN2: Sestrin2

Next, we investigated the effect of metformin on H1299 cell viability. A less than 25% decrease in cell viability was observed in H1299 cells treated with 10 mM metformin for 24 h (Fig. 6A). The combination of kinase inhibitor and biguanide has been reported to have increased antitumour efficacy [30], and we investigated whether lapatinib, a dual EGFR and HER2 kinase inhibitor, enhances cell sensitivity to metformin. Interestingly, lapatinib potentiated the metformin’s effect on the ATF4, REDD1, and Sestrin2 expression and the AMPK phosphorylation (Fig. 6B). Lapatinib enhanced the metformin-induced inhibition of S6K phosphorylation and the inhibitory effect of metformin on the cell viability (Fig. 6B and C). To investigate whether REDD1 and Sestrin2 are involved in cell sensitivity to lapatinib and metformin, we knocked down REDD1 and Sestrin2 in H1299 cells, followed by lapatinib and metformin treatment. siRNA silencing of both REDD1 and Sestrin2 abrogated REDD1 and Sestrin2 expression but did not affect AMPKα phosphorylation induced by metformin treatment (Fig. 6D). Treatment with REDD1 and Sestrin2 siRNA significantly increased viability in cells treated with metformin and lapatinib, suggesting that expression of both REDD1 and Sestrin2 is involved in cell sensitivity to metformin and lapatinib (Fig. 6E). Next, we knocked down ATF4 in H1299 cells, followed by lapatinib and metformin treatment to investigate whether ATF4 is involved in cell sensitivity to lapatinib and metformin. siRNA-mediated knockdown of ATF4 abrogated the expression of ATF4 and its downstream targets REDD1 and Sestrin2 induced by metformin (Fig. 6F). However, ATF4 siRNA did not affect metformin-induced AMPKα phosphorylation (Fig. 6F). ATF4 siRNA significantly increased viability in cells treated with metformin and lapatinib (Fig. 6G). These results suggest that ATF4-mediated REDD1 and Sestrin2 expression is involved in cell sensitivity to metformin and lapatinib.

## Discussion

In the present study, we provide evidence that metformin inhibited mTORC1 signalling independent of AMPK. We found that metformin inhibited mTORC1 signalling via ATF4-induced REDD1 and Sestrin2 expression. Furthermore, we demonstrated that treatment with a combination of metformin and lapatinib significantly reduced the viability of NSCLC cells. siRNAs targeting REDD1 and Sestrin2 significantly increased viability in cells treated with metformin and lapatinib. Our study advances the current understanding of the molecular mechanism used by metformin to regulate mTORC1 pathways as a cancer therapy.

Metformin treatment induced AMPK activation and mTORC1 inhibition (Fig. 1A). It has been reported that metformin requires AMPK to inhibit mTORC1 [26]. In this study, we found that metformin-induced AMPK activation contributed to mTORC1 inhibition. However, downregulation of AMPK did not fully recover metformin-induced mTORC1 inhibition (Fig. 1B). This result suggests that there are additional mechanisms involved in the metformin-mediated inhibition of mTORC1.

REDD1 is one of the best characterized suppressors of mTORC1. REDD1 promotes the association of PP2A with PKB/Akt, ultimately leading to TSC2 activation and mTORC1 inhibition [31]. Sestrin2 has also been reported to inhibit mTORC1 signalling via activation of AMPK [32, 33]. More recently, Sestrin2 was proposed to inhibit mTORC1 through modulation of GATOR complexes [34, 35]. In this study, we found that mTORC1 was partially suppressed by metformin-induced expression of REDD1 or Sestrin2 (Fig. 5A and B). These data suggest that induction of either REDD1 or Sestrin2 alone by metformin cannot completely inhibit mTORC1, and REDD1 and Sestrin2 act together to inhibit mTORC1 following metformin treatment. Interestingly, when compared with control siRNA-treated cells, it was observed that metformin-induced Sestrin2 expression was more elevated in REDD1 siRNA-treated cells, and metformin-induced REDD1 expression was more elevated in Sestrin2 siRNA-treated cells. Further research is needed to confirm in these findings.

It has been reported that metformin increases REDD1 expression in a p53-dependent manner [22]. Because the H1299 cell line used in this study lacks p53, metformin-induced REDD1 expression may be p53-independent. It has been reported that activation of the PERK-eIF2α-ATF4 axis is triggered by metformin [27], and upregulation of REDD1 and Sestrin2 by leucine deprivation is mediated by ATF4 [36]. We found that REDD1 and Sestrin2 induced by metformin are mediated by ATF4. Furthermore, we showed that siRNA targeted against ATF4, REDD1, and Sestrin2 did not change the AMPK activation induced by metformin. Additionally, AMPKα siRNA did not change ATF4-induced REDD1 and Sestrin2 expression. These data suggest that ATF4-induced mTORC1 inhibition by metformin occurs independent of AMPK activation.

The combination of kinase inhibitor and biguanide has been reported to have increased antitumour efficacy [30]. Lapatinib, a dual EGFR and HER2 kinase inhibitor, enhanced the metformin’s effect on the ATF4, REDD1, and Sestrin2 expression and the inhibitory effects of metformin on the viability of H1299 cells. In cells with reduced viability due to combined metformin/lapatinib treatment, treatment with ATF4 siRNA or REDD1/Sestrin2 siRNA significantly increased viability, indicating that ATF4-mediated REDD1 and Sestrin2 expression is involved in cell sensitivity to metformin and lapatinib.

In conclusion, ATF4-mediated REDD1 and Sestrin2 expression triggered by metformin plays an important role in mTORC1 inhibition independent of AMPK activation, and this signalling pathway could have therapeutic value.

## Data Availability

Not applicable.

## Abbreviations

4E-BP1: Eukaryotic initiation factor 4E (eIF4E)-binding protein 1
ACC: Acetyl-CoA carboxylase
AMPK: AMP-activated protein kinase
mTORC1: Mechanistic target of rapamycin 1
NSCLC: Non-small cell lung cancer
Rheb: Ras homology enriched in brain
S6K1: Ribosomal S6 kinase 1
TSC1/2: Tuberous sclerosis complex1/2
