# Correction to: Inhibition of mTORC1 through ATF4-induced REDD1 and Sestrin2 expression by Metformin

**DOI:** 10.1186/s12885-021-08584-z

**Published:** 2021-08-02

**Authors:** Se-Kyeong Jang, Sung-Eun Hong, Da-Hee Lee, Ji-Young Kim, Ji Yea Kim, Sang-Kyu Ye, Jungil Hong, In-Chul Park, Hyeon-Ok Jin

**Affiliations:** 1 Division of Fusion Radiology Research, Korea Institute of Radiological & Medical Sciences, 75 Nowon-ro, Nowon-gu, Seoul, 01812 Republic of Korea; 2 Department of Food and Microbial Technology, Seoul Women’s University, 621 Hwarangro, Nowon-gu, Seoul, 01797 Republic of Korea; 3 KIRAMS Radiation Biobank, Korea Institute of Radiological and Medical Sciences, 75 Nowon-ro, Nowon-gu, Seoul, 01812 Republic of Korea; 4 Department of Pharmacology, Seoul National University College of Medicine, 103 Daehak-ro, Jongno-gu, Seoul, 03080 Republic of Korea

**Correction to: BMC Cancer 21, 803 (2021).**

10.1186/s12885-021-08346-x

Following publication of the original article [1], the authors reported that the electrophoretic blots of Figs. 1A, B, 2A, C, E, 3A, C, E, 4A, B, C, D, 5A, B, C, 6B, D, and F were published in their raw format. The edited versions of the complete figures are given below and the original article [1] has been corrected.

**Fig. 1:**
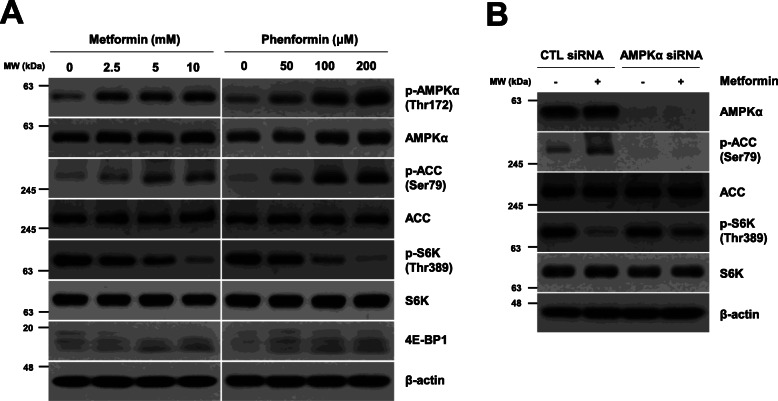
Metformin inhibits mTORC1 through AMPK. (a) H1299 cells were treated with the indicated concentrations of metformin or phenformin for 24 h. (b) H1299 cells were transfected with control or AMPKα siRNA for 12 h and were then treated with 10 mM metformin for 24 h. (a, b) Data are representative of two independent experiments. CTL: control

**Fig. 2:**
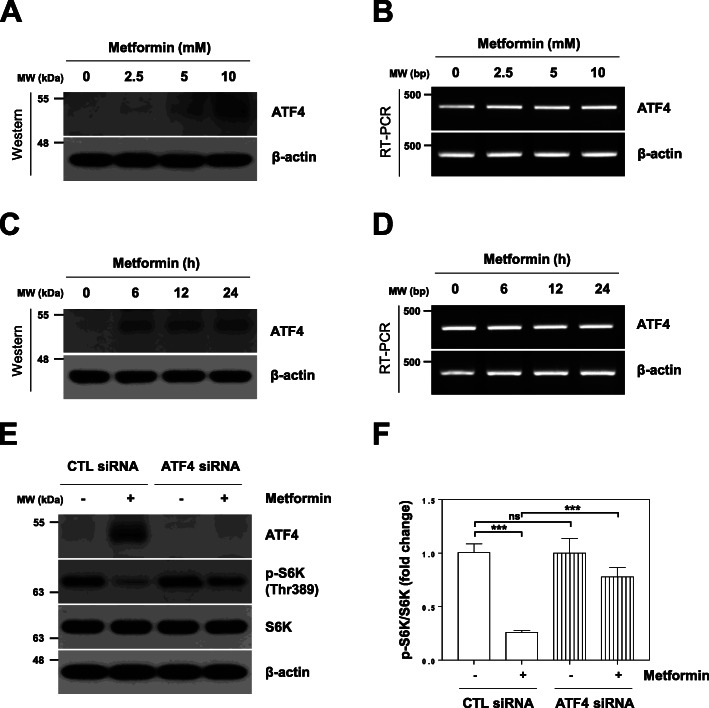
Metformin inhibits mTORC1 through ATF4. (a, b) H1299 cells were treated with the indicated concentrations of metformin for 24 h. (c, d) H1299 cells were treated with 10 mM metformin for the indicated time. The protein levels (a, c) and mRNA levels (b, d) were estimated by western blot and RT- PCR analysis, respectively. (a-d) Data are representative of two independent experiments. (e, f) H1299 cells were transfected with control or ATF4 siRNA for 12 h and were then treated with 10 mM metformin for 24 h. (e) Data are representative of three independent experiments. (f) The p-S6K expression was quantified using ImageJ software and fold change with respect to control after normalization to respective S6K bands was plotted as histogram. (*n* = 3; ***P < 0.001; NS, not significant). CTL: control

**Fig. 3:**
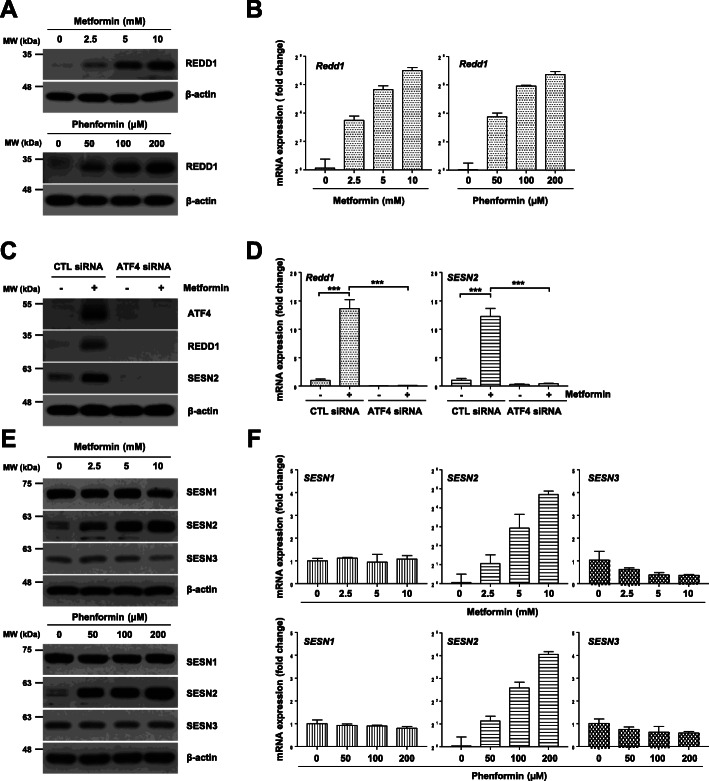
Metformin induces the expression of REDD1 and Sestrin2 in an ATF4-dependent manner. (a, b, e, f) H1299 cells were treated with the indicated concentrations of metformin or phenformin for 24 h. (c, d) H1299 cells were transfected with control or ATF4 siRNA for 12 h and were then treated with 10 mM metformin for 24 h. The protein levels (a, c, e) and mRNA levels (b, d, f) were estimated by western blot and real-time PCR analysis, respectively. (a, c, e) The western blot is representative of two independent experiments. (b, d, f) The real-time PCR results for each sample were analysed according to the 2^−ΔΔCt^ method using β-actin as the internal control. Gene transcription is presented as the fold change relative to the control sample (*n* = 3; ***P < 0.001). CTL: control, SESN1: Sestrin1, SESN2: Sestrin2, SESN3: Sestrin3

**Fig. 4:**
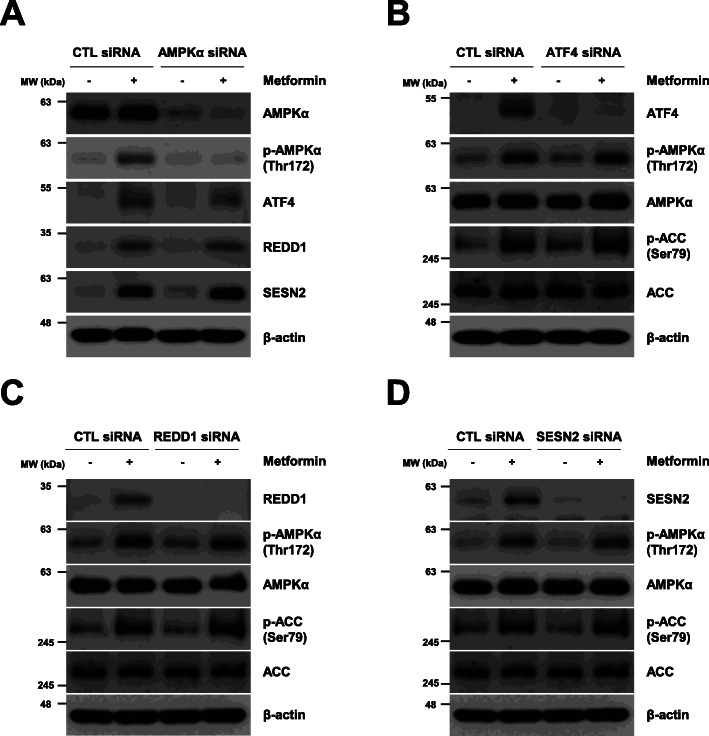
AMPK and ATF4 do not affect each other’s expression under metformin treatment. (a–d) H1299 cells were transfected with control, AMPKα, ATF4, REDD1, or Sestrin2 siRNA for 12 h and then treated with 10 mM metformin for 24 h. The indicated protein levels were estimated by western blot analysis. The blot is representative of two independent experiments. CTL: control, SESN2: Sestrin2

**Fig. 5:**
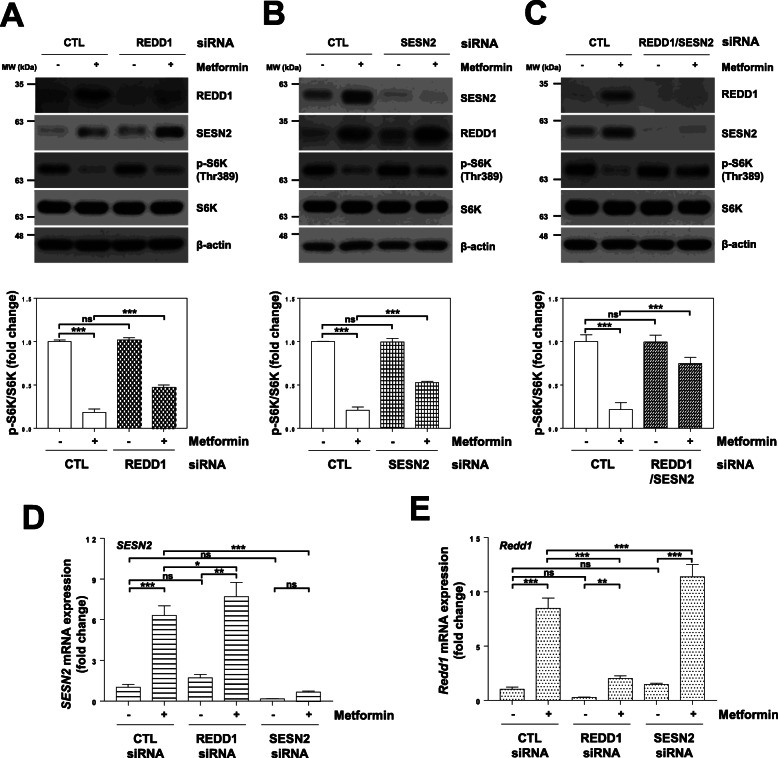
Metformin-induced REDD1 and Sestrin2 expression is involved in mTORC1 inhibition by metformin. (a-e) H1299 cells were transfected with control, REDD1, Sestrin2, or REDD1/Sestrein2 siRNA for 12 h and then treated with 10 mM metformin for 24 h. (a, b, c) The indicated protein levels were estimated by western blot analysis. (upper panels; a, b, c) The blot is representative of three independent experiments. (bottom panels; a, b, c) The p-S6K protein expression was quantified using ImageJ software and fold change with respect to control after normalization to respective S6K protein bands was plotted as histogram (*n* = 3; ***P < 0.001; ns, not significant). (d, e) The indicated mRNA levels were estimated by real-time PCR analysis. The real-time PCR results for each sample were analysed according to the 2^−ΔΔCt^ method using β-actin as the internal control. Gene transcription is presented as the fold change relative to the control sample (*n* = 3; *p < 0.05; **p < 0.01; ***p < 0.001; ns, not significant). CTL: control, SESN2: Sestrin2

**Fig. 6:**
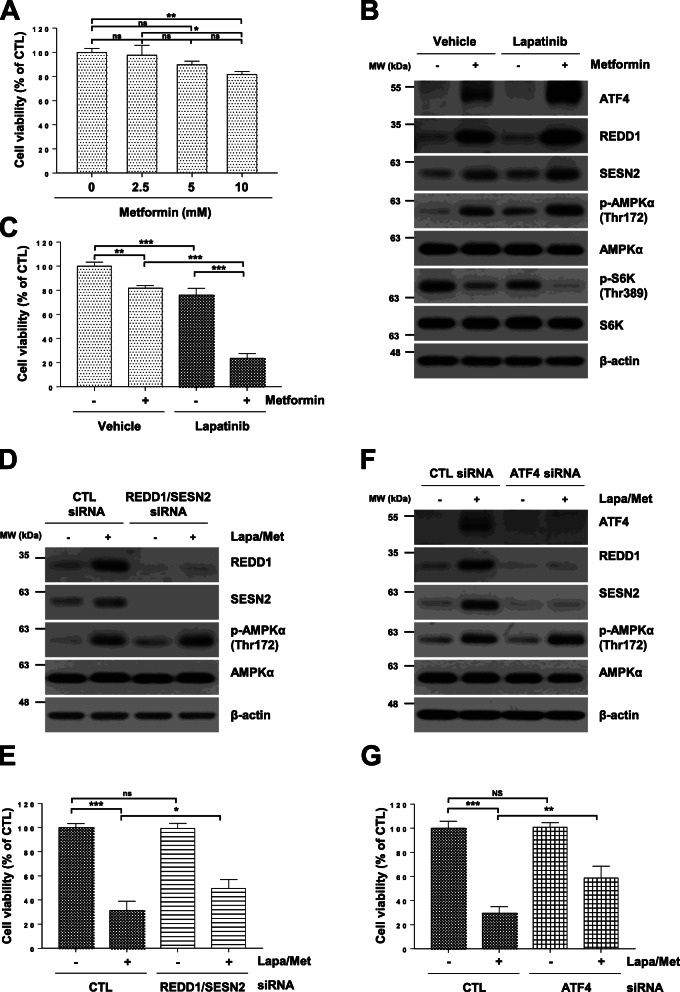
Lapatinib enhances cell sensitivity to metformin, and knockdown of REDD1 and Sestrin2 decreases cell sensitivity to metformin and lapatinib. (a) H1299 cells were treated with the indicated concentrations of metformin for 24 h. (b) H1299 cells were treated with 10 mM metformin and/or 10 μM lapatinib for 12 h. (c) H1299 cells were treated with 10 mM metformin and/or 10 μM lapatinib for 24 h. (d, f) H1299 cells were transfected with control, ATF4 or REDD1/Sestrin2 siRNA for 12 h followed by treatment with 10 mM metformin and 10 μM lapatinib for 12 h. (e, g) H1299 cells were transfected with control, ATF4, or REDD1/Sestrin2 siRNA for 12 h followed by treatment with 10 mM metformin and 10 μM lapatinib for 24 h. (a. c, e, g) Cell viability was measured by MTT assay. The data are presented as the mean percentage of control ± SD relative to the control (*n* = 3; *p < 0.05; **p < 0.01; ***p < 0.001; ns, not significantly different). (b, d, f) The indicated protein levels were estimated by western blot analysis. Data are representative of three independent experiments. CTL: control, Lapa: Lapatinib, Met: metformin, SESN2: Sestrin2
